# The Effect of Locomotion on Early Visual Contrast Processing in Humans

**DOI:** 10.1523/JNEUROSCI.1428-17.2017

**Published:** 2018-03-21

**Authors:** Alex V. Benjamin, Kirstie Wailes-Newson, Anna Ma-Wyatt, Daniel H. Baker, Alex R. Wade

**Affiliations:** ^1^Department of Psychology, University of York, York YO105DD, United Kingdom and; ^2^Department of Psychology, University of Adelaide, Adelaide, South Australia, 5005 Australia

**Keywords:** arousal, gain control, locomotion, murine models, SSVEP

## Abstract

Most of our knowledge about vision comes from experiments in which stimuli are presented to immobile human subjects or animals. In the case of human subjects, movement during psychophysical, electrophysiological, or neuroimaging experiments is considered to be a source of noise to be eliminated. Animals used in visual neuroscience experiments are typically restrained and, in many cases, anesthetized. In reality, however, vision is often used to guide the motion of awake, ambulating organisms. Recent work in mice has shown that locomotion elevates visual neuronal response amplitudes (Niell and Stryker, 2010; Erisken et al., 2014; Fu et al., 2014; Lee et al., 2014; Mineault et al., 2016) and reduces long-range gain control (Ayaz et al., 2013). Here, we used both psychophysics and steady-state electrophysiology to investigate whether similar effects of locomotion on early visual processing can be measured in humans. Our psychophysical results show that brisk walking has little effect on subjects' ability to detect briefly presented contrast changes and that co-oriented flankers are, if anything, more effective masks when subjects are walking. Our electrophysiological data were consistent with the psychophysics indicating no increase in stimulus-driven neuronal responses while walking and no reduction in surround suppression. In summary, we have found evidence that early contrast processing is altered by locomotion in humans but in a manner that differs from that reported in mice. The effects of locomotion on very low-level visual processing may differ on a species-by-species basis and may reflect important differences in the levels of arousal associated with locomotion.

**SIGNIFICANCE STATEMENT** Mice are the current model of choice for studying low-level visual processing. Recent studies have shown that mouse visual cortex is modulated by behavioral state: primary visual cortex neurons in locomoting mice tend to be more sensitive and less influenced by long-range gain control. Here, we tested these effects in humans by measuring psychophysical detection thresholds and electroencephalography (EEG) responses while subjects walked on a treadmill. We found no evidence of increased contrast sensitivity or reduced surround suppression in walking humans. Our data show that fundamental measurements of early visual processing differ between humans and mice and this has important implications for recent work on the links among arousal, behavior, and vision in these two species.

## Introduction

Recent work in head-fixed mouse models has demonstrated that locomotion is linked with changes in early visual processing. Many studies have reported that locomoting mice exhibit increased responsivity in primary visual cortex (V1) (Niell and Stryker, 2010; Polack et al., 2013; Fu et al., 2014), whereas there is also evidence for a locomotion-associated reduction in surround suppression (Ayaz et al., 2013) and locomotion-dependent visual plasticity (Kaneko and Stryker, 2014; Kaneko et al., 2017). These measurements are broadly consistent with the more general observations that sensory neuronal responses are dependent, not just on stimulus strength, but also on behavioral state, arousal, and attention (Posner and Petersen, 1990; Motter, 1993; Lauritzen et al., 2010; Harris and Thiele, 2011; Haider et al., 2013; Reimer et al., 2014; McGinley et al., 2015). However, the underlying mechanisms linking locomotion to visual sensitivity in mice are unclear, as are the implications for human vision. Some investigators have reported modulations of early human visual processing during periods of acute exercise changes, but these are at the level of featural tuning (Bullock et al., 2017), whereas the effects on low-level contrast sensitivity are more ambiguous (Bullock et al., 2015). Moreover, these effects are observed, not during locomotion per se, but rather during intense bouts of exercise on a stationary bicycle. To our knowledge, the most striking effect of true locomotion on human vision to date has been the observation of a locomotion-related motion aftereffect the cause of which has never been fully explained (Pelah and Barlow, 1996) but which must act at a level above simple contrast processing in V1.

If locomotion alters early contrast representations in humans, then it would have profound implications for our understanding of natural scene processing. Orientation-selective surround suppression (Nelson and Frost, 1978; DeAngelis et al., 1994; Cavanaugh et al., 2002) has been hypothesized to play a critical role in scene segmentation by increasing neuronal responses at the boundaries of different texture patches (Knierim and van Essen, 1992; Lamme, 1995; Nothdurft et al., 2000; Rossi et al., 2001). The discovery of a significant reduction in surround suppression during locomotion would therefore raise the possibility that scene segmentation is altered (and potentially impaired) while subjects are navigating their environment. Similarly, a locomotion driven change in neuronal gain would reshape or reposition the contrast sensitivity function, with implications for the discrimination of both low- and high-contrast edges and the computation of speed, which is known to be contrast dependent (Thompson, 1982; Stocker and Simoncelli, 2006).

Here, we measured two aspects of early contrast processing, neuronal sensitivity and surround suppression, in locomoting humans. These measurements were made using two sensitive and complementary methods, psychophysical contrast discrimination and steady-state EEG, to provide both perceptual and direct neuronal measures of contrast processing. The locomotion of the participants (on a treadmill) was varied across repetitions of the experiment. We then investigated whether we were able to measure changes in either responsivity or orientation-dependent surround suppression between the locomotion and static conditions. We compare our findings with those from the mouse literature, with particular reference to the interaction between arousal and locomotion states in humans and mice.

## Materials and Methods

### 

#### 

##### General experimental design.

We performed behavioral and electrophysiological (steady-state visually evoked potential, SSVEP) experiments to measure neuronal response amplitude and long-range, spatially tuned gain control in human subjects. Thirteen subjects (4 female, mean age 26) took part in the behavioral experiment, 13 subjects (10 female, mean age 24) took part in the SSVEP experiments, and 12 subjects (8 female, mean age 24) took part in the pupillometry experiment. Nine subjects took part in all experiments. All experimental protocols were approved by the ethics committee of the University of York Psychology Department.

All measurements were collected under two conditions: a “locomotion” or “walking” condition, in which subjects walked on a motorized treadmill, and a “static” condition, in which they straddled the moving treadmill belt (width = 60 cm). Psychophysical subjects also participated in a third “target moves” condition to test the potential effects of retinal motion.

The same treadmill (GTR Power Pro; Confidence Fitness) was used in all experiments and ran constantly at a preset speed of 5 km/h, which is equivalent to a brisk walk.

##### Experiment 1: psychophysics.

Stimuli were presented on a Multisync CRT monitor (Mitsubishi) running at 100 Hz under the control of an OSX 10.9 computer (Apple) running Psykinematix version 1.4 software (Kybervision). The monitor was positioned at a distance of 110 cm from the subjects and centered vertically at face level. Spectral and gamma calibration was performed using a Spyder4 colorimeter (Datacolor) and cross-checked with a fiber-optic photospectrometer (Jaz; Oceanoptics). All stimuli were presented on a mean-gray background with a luminance of 94 cd/m^2^. Responses were registered using an OSX-compatible USB gamepad (Logitech) fixed to the handle of the treadmill.

Subjects performed a set of contrast discrimination/detection judgements using stimuli similar to those described previously (Wade, 2009; Petrov et al., 2005). A pair of “probe” Gabor patches (σ = 1.5°, spatial frequency = 2 cpd) were presented simultaneously for 200 ms 5° to the left and right of a fixation marker. One of the probes had a “pedestal” contrast C and the other had a contrast C+ΔC. The subject's task was to indicate which probe (left or right) had the higher contrast. For each pedestal level (0%, 1%, 2%, 5%, and 10%), the magnitude of ΔC was determined using a Bayesian adaptive staircase procedure (Kontsevich and Tyler, 1999) to obtain a threshold at 78% correct. Staircases for all pedestal levels were interleaved and six repetitions of each threshold were obtained for each subject. Motion conditions (walking/stationary/target moves) were interleaved at random and each condition lasted ∼9 min.

To eliminate uncertainty about the spatial location of the probes (Petrov et al., 2006), a thin gray circle was present around the probe locations throughout the experiment. Similarly, to eliminate uncertainty about the temporal location of the stimuli, their onset was cued by a subtle change in the shape of the fixation point 200 ms before stimulus onset. Subjects received audio feedback (high or low tones to indicate correct or incorrect responses) throughout the experiments.

**Figure 1.**
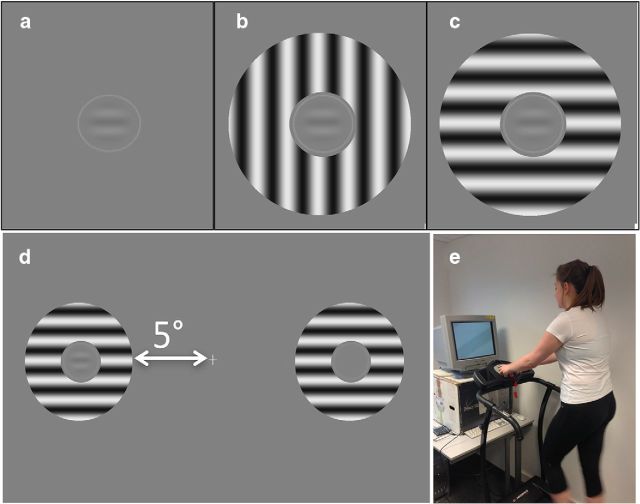
Stimulus configurations. ***a***, No mask; ***b***, Orthogonal mask. ***c***, Collinear mask. Stimuli were presented in a spatial 2AFC paradigm at ±5° from fixation for 200 ms at a time (***d***). Subjects indicated the position of the central probe with the highest contrast while either standing on a powered treadmill (***e***) or straddling the active treadmill belt.

To measure the effects of surround suppression, we measured thresholds for isolated probes and also for probes placed in the center of annular “surrounds” containing high-contrast (90%) gratings. A gap of one grating wavelength (1λ) was present between the probe and the surround to minimize the contribution of isotropic precortical “overlay masking” (Petrov et al., 2005) and the outer radius of the annulus was 6°. Because cortical surround suppression is tuned for orientation, we measured the effects of surround gratings in two configurations: collinear and orthogonal with the probe Gabor (Fig. 1).

In addition to the “locomoting” and “static” conditions, a third “static/target moving” or “s/tm” condition was generated in an attempt to simulate the effects of locomotion on retinal image position. In this “s/tm” condition, both sets of probe + surround drifted rapidly (30°/s) in the same randomly chosen direction for the duration of the 200 ms presentation. We included this condition as a conservative test of the effect of retinal image motion and blurring. In total, we measured discrimination/detection thresholds for 15 different combinations of surround type (3) and contrast (5) for each of three locomotion conditions.

##### Experiment 2: SSVEPs.

The stimuli used in the SSVEP experiment were conceptually similar to those used in Experiment 1 but modified to optimize the evoked neuronal signal. Stimuli were generated in using the Psychophysics toolbox running on an OSX 10.10 computer (Apple) and displayed on a calibrated ViewPixx monitor (VPixx Technologies) running at a frame rate of 120 Hz with a mean background luminance of 84 cd/m^2^.

The “probe” Gabors had a spatial frequency of 2 cpd and a diameter of 1.2°, windowed by a raised cosine envelope. These frequency-tagged probes were presented at a range of fixed contrast levels with three types of surround (no surround, collinear surround, and orthogonal surround). The probes appeared and disappeared (on/off) at a fixed frequency (7 Hz sinusoidal flicker) and therefore generated a phase-locked response at 7 Hz in the EEG record over visual cortex, with additional second harmonic transients at 14 Hz. When present, the high-contrast sine wave grating surround (96% contrast, 2 cpd) drifted at a speed of 3°/s. Drifting gratings are effective surround masks (Xiao and Wade, 2010), but do not generate a coherent frequency-locked response in SSVEP (Norcia et al., 2015).

**Figure 2.**
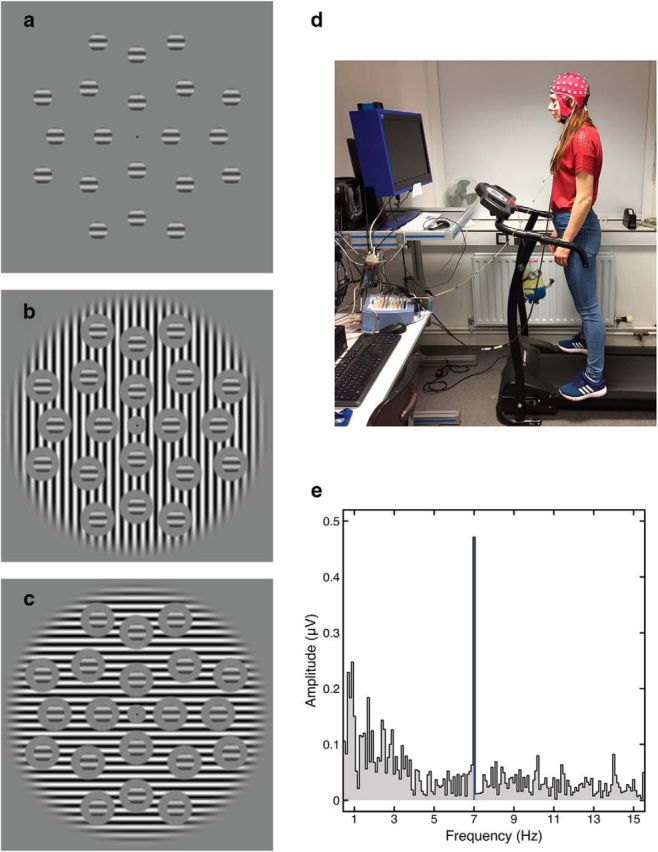
Example stimuli, photograph of experimental set-up, and example Fourier spectrum. ***a***, Matrix of target stimuli, which were rotated about the central fixation by a random amount on each trial. ***b***, Target stimuli with an orthogonal surround mask. ***c***, Target stimuli with a collinear surround mask. The phase alignment between target and mask is arbitrary because the drifting mask meant that the relative phases of the two stimuli changed over time. ***d***, Photograph of the experimental setup, including the treadmill and a participant wearing an EEG cap. ***e***, Example Fourier spectrum taken from the stationary condition for the highest target contrast tested with no mask. A strong, well isolated response is evident at the target frequency of 7 Hz.

To maximize the EEG response, multiple probe patches (*n* = 20) were present on screen at any moment, arranged in a hexagonal grid with a diameter of 20° (Fig. 2*a*). Absolute stimulus orientation was randomized on each trial to avoid local adaptation aftereffects, but the relative orientation of target and surround was controlled according to condition (collinear or orthogonal). The offset between the edge of the target gratings and the inner edge of the mask was one full grating cycle (0.5°).

EEG data were recorded at 1 kHz using an ANT Neuroscan EEG system with a 64-channel Waveguard cap. Stimulus onset was recorded on the EEG trace using low-latency digital triggers sent over a parallel cable from the ViewPixx device. The first 1 s of each 11 s trial was discarded to remove onset transients and a fast Fourier transform was taken of the EEG trace from the remaining 10 s, giving a frequency resolution of 0.1 Hz. We performed coherent averaging across trials within a condition for each participant and then averaged the absolute amplitude values across participants. To calculate signal-to-noise ratios (SNRs), we averaged the amplitudes in the 10 frequency bins adjacent to the signal frequency (from 6.5–6.9 Hz and from 7.1–7.5 Hz in 0.1 Hz steps) and divided the amplitude in the signal bin by this average.

As in the psychophysical experiments, responses were recorded under two randomized, interleaved conditions: “static” and “locomoting” (brisk walking at 5 km/h) in blocks of ∼9 min.

##### Experiment 3: pupillometry.

Systemic arousal in both humans and mice can be correlated with both neurophysiological and behavioral changes (Bradley et al., 2008; Murphy et al., 2011; McGinley et al., 2015). To measure the effects of treadmill walking on arousal, we used a head-mounted, infrared-illuminated, video-based eye tracker (Pupil Laboratories) to measure pupil sizes in subjects (*n* = 12) performing the psychophysical task in both stationary and walking conditions in a randomized order using room illumination conditions identical to those in Experiment 1. The eye tracker software “Pupil Capture” collected 10 min of samples at 120 Hz and pupil size and confidence measures for both left and right eye were recorded. Data from the first half of each measurement block were discarded to remove artifacts due to residual light adaptation and mechanical “settling” of the eye tracker on the head. A separate measurement was conducted to measure maximum pupil size in perceptual darkness (with infrared pupil illuminations) to ensure that the pupil was not fully dilated in the psychophysics task under dim illumination.

Measurements were analyzed offline using MATLAB (The MathWorks) and R (R Development Core Team, 2008) and only pupil diameters with a confidence rating >0.95 (maximum = 1) were retained. Because the absolute mean pupil size depends on many factors, including the angle of the eye-tracking camera and the proximity to the head, we present all data in units of screen pixels and assess the difference between walking and stationary conditions. We performed within-subjects *t* tests on raw pupil diameter measures from left and right eyes independently and a paired *t* test on the entire group.

##### Statistical analyses.

We fit our psychophysical and neurophysiological data assuming an underlying neuronal response function that has the form of a hyperbolic ratio function (see Eq. 3 in Albrecht and Geisler, 1991).

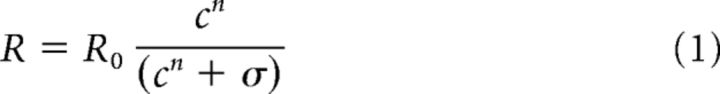
 In the case of our psychophysical data, we assumed that the thresholds were proportional to the first derivative of this hyperbolic ratio function that we computed analytically. This model is common in the psychophysical literature and rests on the assumption that detection or discrimination is limited by a single, late noise source (Nachmias and Sansbury, 1974; Boynton et al., 1999; Itti et al., 2000). In the case of the neuronal data, we fit the parameters of the hyperbolic ratio function directly.

**Figure 3.**
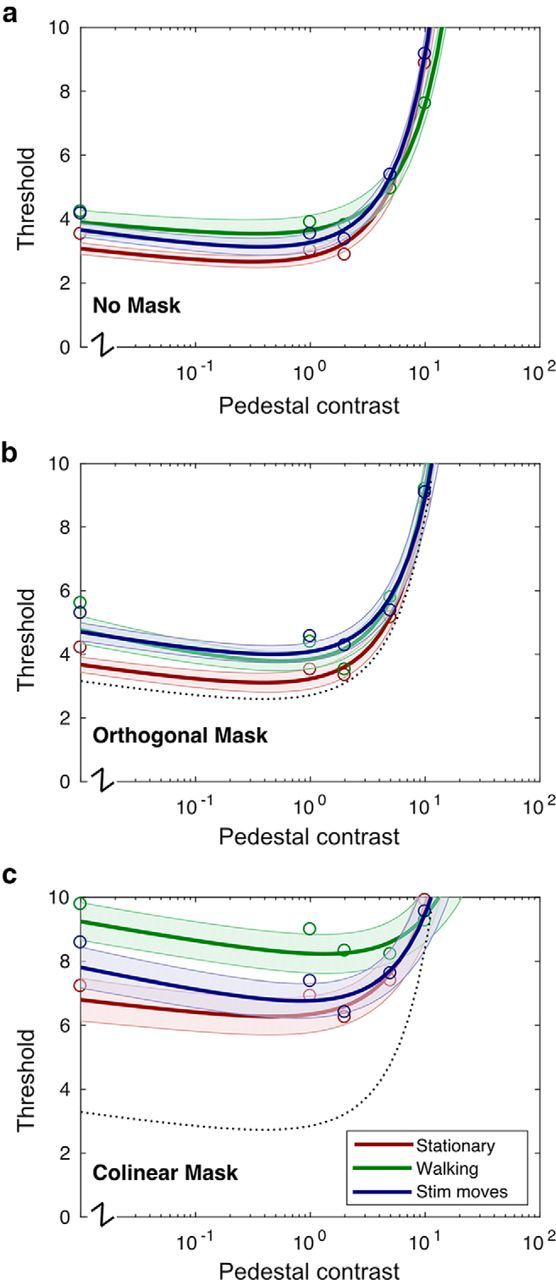
Detection/discrimination thresholds measured at five different pedestal levels. Orthogonal masks (***b***) generate almost no change in threshold compared with the unmasked condition (***a***), whereas collinear masks (***c***) raise thresholds significantly. Collinear masking is significantly higher in the walking (green) condition. Unmasked/stationary thresholds are replotted as dashed black lines in ***b*** and ***c*** for comparison.

**Figure 4.**
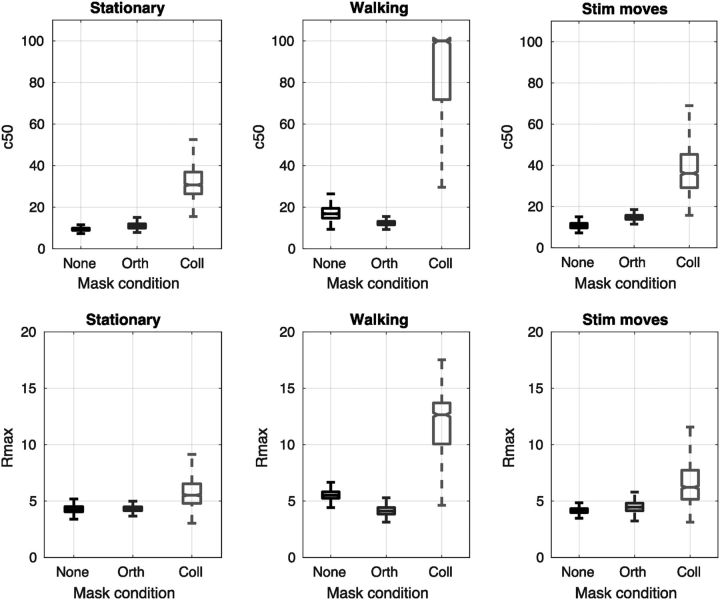
Bootstrapped parameters for hyperbolic ratio functions fitted to psychophysical data. Locomotion causes a significant increase in the *c*_50_ and a small but still significant increase in the *R*_max_. Notches indicate 95% confidence intervals.

To obtain error bounds for our fits and to avoid the use of parametric statistics, we used permutation methods to bootstrap the model parameters by resampling data points from our 13 subjects with replacement and recomputing model fits a total of 10,000 times (Efron and Tibshirani, 1993) using the MATLAB function *bootci*. The error bounds shown in Figures 3 and 6 are derived from these bootstraps and indicate the 95% confidence intervals. Similarly, in Figures 4 and 7, the boxplots show the range of the bootstrapped parameters with the notches indicating the 95% confidence intervals.

##### Sample sizes.

Niell and Stryker (2010) reported that motion increased population activity by ∼300% both for spontaneous gamma power and for measures of individual stimulus-driven neuronal responses (spikes/s). If such large effects were present in our EEG data (in which we also measured neuronal responses to high-contrast gratings), then we would expect to measure significant (*p* < 0.001) walking-driven SNR differences for the high-contrast, unmasked probes with a sample size of no more than three subjects, even assuming a twofold increase in overall noise (Lenth, 2001; Rosner, 2011). Ayaz et al. (2013) reported a more modest reduction in the amount of surround suppression that they measured in locomoting animals. Their population average suppression index (defined as the normalized difference in response between an optimal stimulus and one suppressed by the surround) decreased by a factor of ∼40% (from 38% to 23%) when their mice were locomoting.

We acknowledge that the relationship between population average responses of neuronal activity as measured by single units and scalp-level EEG is not direct, but nevertheless, we observed that our EEG measurements of *R*_max_ were reduced by ∼25% between static/unmasked and static/suppressed, suggesting that our baseline suppression index would be comparable to that seen in the Ayaz et al. (2013) study. Again, using realistic estimates of noise, we calculated that we would require no more than four subjects to detect this level of change at the *p* < 0.001 level and we estimate that our actual sample sizes (13 subjects) had enough power to identify effects less than half the size of the magnitudes reported in the single-unit literature.

## Results

### Experiment 1: psychophysics

Figure 3 shows threshold data for all combinations of locomotion condition and surround type. Thresholds for the unmasked condition are shown in Figure 3*a*. These exhibit a classic “dipper” shape (Nachmias and Sansbury, 1974; Foley and Legge, 1981), with the lowest threshold occurring at a pedestal level of approximately half the detection threshold. Thresholds in the stationary condition (red line) are slightly lower than the other two conditions; for example, probe detection thresholds (zero pedestal) in the “no mask” condition increased from 3.8% to 4.2% (*p* < 0.001) when subjects were walking. However, in general, unmasked thresholds for the “stationary,” “walking,” and “stimulus moves” conditions are strikingly similar, suggesting that subjects are able to perform the task well under all conditions, that walking per se does not impose a significant attentional or fixational penalty, and that in this experiment, subjects can compensate for relatively large amounts of retinal motion (Westheimer and McKee, 1975). Walking also does not appear to increase sensitivity to unmasked targets, which might be expected to lead to reduced thresholds or a leftward shift in the curve.

Figure 3*b* shows thresholds measured for the “orthogonal mask” condition. The unmasked, stationary thresholds are replotted as a dotted line for reference. Thresholds are slightly elevated in this condition, but the effects are small and consistent with those seen in other studies of surround suppression (Petrov et al., 2005).

Figure 3*c* shows thresholds measured in the “collinear mask” condition, in which targets are suppressed by a cooriented annular surround. These thresholds are significantly higher than those measured in either the “no mask” or “orthogonal mask” conditions, consistent with the idea that we are measuring a suppressive, long-range, orientation-tuned (and therefore cortical) phenomenon.

Detection/discrimination thresholds measured during the conlinear locomotion condition (Figure 3*b*, green line) are higher, not lower, than those measured when subjects are either stationary or viewing moving targets (red, blue lines). In brief, walking appears to increase, not decrease, psychophysical surround suppression. Although unmasked thresholds are also slightly higher in the “locomoting” condition, surround suppression is also increased significantly by walking when the effect is computed as a multiple of the unmasked threshold contrast.

Figure 4 shows the bootstrapped parameter fits for *c*_50_ (the semisaturation constant) and *R*_max_ (the maximum amplitude) under different surround and locomotion conditions. Interestingly, estimates of both parameters are significantly larger for the walking collinear condition than for the stationary moves or target moves collinear conditions. This indicates that, although the suppressive effects of contrast gain control appear to be, if anything, amplified in the walking condition (i.e., *c*_50_ is larger, implying that sensitivity is reduced), response gain (as measured by *R*_max_) may also be altered in a manner that increases the maximum response level of the neuronal population at the highest contrast levels.

### Experiment 2: SSVEP

**Figure 5.**
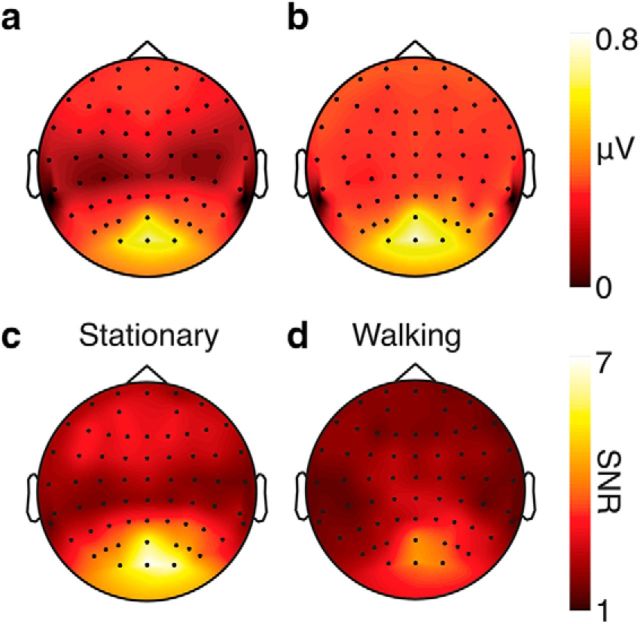
Grand average responses at the first harmonic of the stimulus modulation rate for isolated (unmasked) probes. ***a***, ***b***, Raw amplitude at the tag frequency F1. ***c***, ***d***, Ratio of F1 to the average amplitude of the local side bins (SNR). Although raw amplitude is higher in the locomotion condition, this is due to an increase in broadband noise and not an increase isolated to the SSVEP signal frequency.

Figure 5 shows the average response to unmasked probes combined across all subjects. As expected, the dominant response is centered on Oz consistent with a source in early visual cortex. Figure 5, *a* and *b*, show the raw response amplitudes in the stationary and locomotion conditions, respectively. Amplitudes are higher overall in the locomotion condition, but this could reflect either a higher neuronal response restricted to the stimulus frequency or a generally increased response in the EEG signal due to broadband noise. Figure 5, *c* and *d*, show SNR rather than raw amplitude and confirm that SNR drops in the locomoting condition compared with the stationary condition. There is therefore no evidence that active walking increases neuronal responses to the frequency-tagged probe.

**Figure 6.**
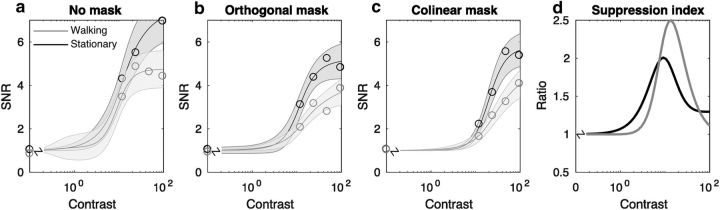
SNR ratios as a function of stimulus contrast under different mask conditions: no mask (***a***), orthogonal mask (***b***), collinear mask (***c***), and suppression index (***d***). Surrounds cause a reduction in sensitivity (increase in *c*_50_) and *R*_max_, with the collinear surround generating the largest changes. SNR is lower overall in the walking condition due to an increase in broadband noise. ***d***, Suppression index computed as the ratio of the SNRs in the “no mask” and “collinear mask” conditions. There is no evidence of an increase in raw signal SNR (***a***) and no evidence of a reduction in tuned surround suppression (***c***) in the locomoting condition (***d***).

**Figure 7.**
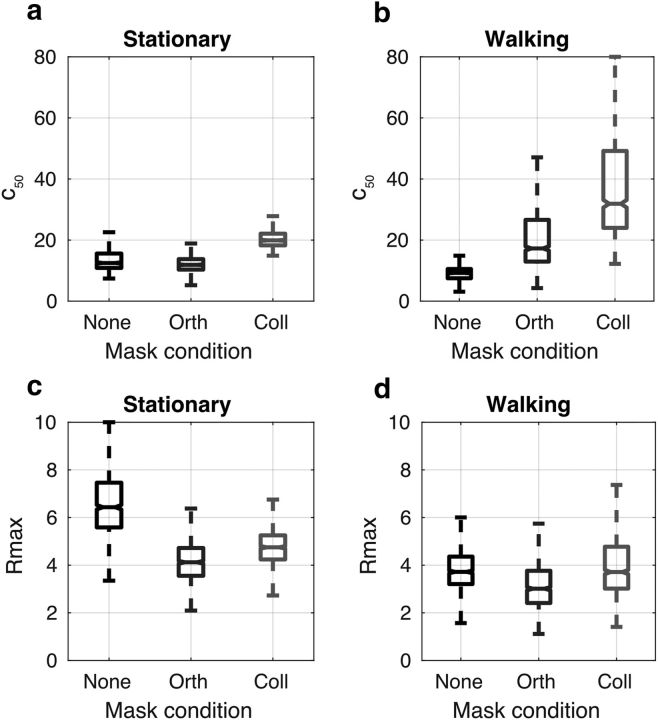
Parameter fits for SSVEP contrast response functions. In the stationary condition (***a***), orientation-tuned surround suppression increases *c*_50_ (reducing sensitivity). In the walking condition, this effect is increased (***b***). Overall, *R*_max_ is reduced slightly in the walking/locomotion condition (***d***). Compared to the stationary condition (***c***).

Figure 6 shows hyperbolic contrast response functions of the form described in Equation 1 fitted to the population SNR data from all 13 subjects with bootstrapped 95% error bounds. Consistent with the data from Figure 5, overall SNR is lower in the locomoting condition (quantified in the fits below). Both conditions show evidence of orientation tuned surround suppression: the lines in Figure 6*c* tend to lie to the right and below of the corresponding lines in Figure 6*a*. There is no overt reduction in the size of the surround suppression during the locomoting condition; if anything, the suppression index (computed as the ratio of SNRs in the unmasked and collinear mask conditions) is higher for walking than for stationary observers on average (Fig. 6*d*). This was confirmed by examining the distribution of the bootstrapped fit parameters (Fig. 7): The semisaturation constant *c*_50_ for unmasked probes is very similar to that computed for psychophysical data, ∼10%, suggesting that our EEG measurements provide a reliable estimate of behavioral sensitivity. It is not possible to compare *R*_max_ values in the psychophysical and SSVEP experiments due directly to the change in measurement units. Evidence of orientation-tuned surround suppression is provided by the fact that the *c*_50_ for collinear surrounds is reliably higher than for the unmasked stimulus or orthogonally masked stimulus for both stationary and locomoting conditions. Consistent with the psychophysical data, collinear-masked *c*_50_ is higher in the locomoting condition than it is in the static condition (*p* < 0.001), not lower, as we would expect if surround suppression were reduced. *R*_max_ also shows a statistically significant reduction overall (*p* < 0.001) in the locomoting condition, indicating that the SNR had not improved overall (see Discussion).

### Experiment 3: pupillometry

**Figure 8.**
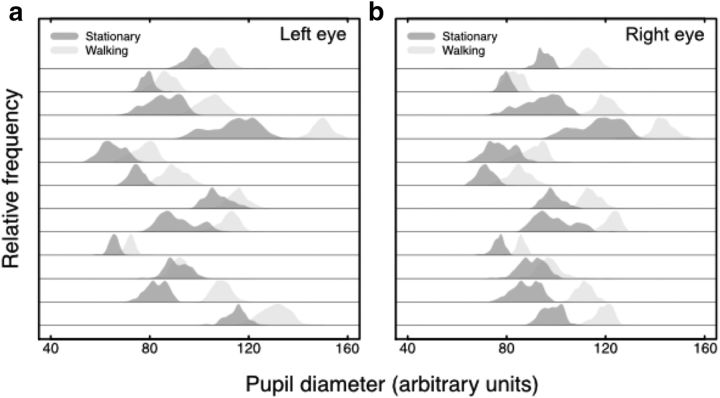
Pupil diameters measured in stationary (dark gray) and walking (light gray) conditions. Data from left and right eyes are plotted separately in ***a*** and ***b*** and each row shows data from a different subject. All subjects had larger pupil diameters in the walking condition (mean diameter increase of 16%, area increase of 34%, *p* < 0.001).

Pupil sizes measured in both eyes were significantly larger (35% increase in area on average, *p* < 0.001) in the walking compared with the stationary conditions (Fig. 8). This size increase was not an artifact of increased noise generated by head movement during locomotion: we explicitly chose only measurements from frames with a high confidence rating (>95%) indicating an error-free fit while visual inspection of individual frames showed no evidence of motion blur or distortion. Similarly, task difficulty (as assessed by raw unmasked detection thresholds) was not significantly greater in the walking compared with the stationary condition (Fig. 4).

## Discussion

We studied the effects of locomotion on long-range, orientation-tuned gain control using both behavioral and electrophysiological methods. The data from the locomotion condition clearly differed from those collected under static conditions, but we saw no evidence for an increase in either spontaneous firing rate or sensitivity when walking. Instead, we measured very little effect of walking on detection/discrimination thresholds when targets were unmasked or surrounded by an orthogonal grating and significantly increased thresholds in the presence of a collinear surround. Our EEG data were equally clear: walking reduced the SNR of our responses slightly overall (possibly due to the introduction of broadband noise) and sensitivity (as measured by *c*_50_) decreased significantly for collinear-masked targets and, to some extent, for targets with orthogonal masks, whereas the responses to unmasked targets were essentially unchanged. Walking seemed to have little effect on unmasked sensitivity and increased, rather than decreased, surround suppression in both experiments.

Robust changes in cortical visual sensitivity linked to locomotion have been measured in mice (Niell and Stryker, 2010; Ayaz et al., 2013; Polack et al., 2013; Saleem et al., 2013; Fu et al., 2014; Lee et al., 2014; Reimer et al., 2014). Although locomotion does not affect responses in the LGN or input layers (Niell and Stryker, 2010), neurons in layer 2/3 of mouse visual cortex are relatively depolarized during locomotion (Polack et al., 2013), leading to higher spontaneous firing rates and increased visual sensitivity. One potential mechanism is that locomotion acts in a top-down manner through a two-layer network regulating visual gain control: stimulating neurons that subsequently inhibit a second class of inhibitory interneurons (Pfeffer et al., 2013; Fu et al., 2014). The same mechanism may contribute to the finding that the suppressive effects of extraclassical receptive fields are also reduced in locomoting animals (Ayaz et al., 2013).

Recent work has also shown that locomotion and arousal are usually tightly coupled in mice: high levels of arousal in mice often induce running behavior and running mice tend to be highly aroused. When the physiological effects of arousal are isolated, it can be shown that arousal that leads to an increase in neuronal sensitivity (Reimer et al., 2014; McGinley et al., 2015) even in the absence of locomotion. In support of this, recent work by Vinck et al. (2015) has shown specifically that sensitivity increases in mouse visual cortex due to arousal can be dissociated from an increase in baseline firing rate due to locomotion.

Our failure to find robust increases in neuronal sensitivity in locomoting humans might be explained by the behavioral and cognitive differences between people and mice. Humans are not necessarily aroused by brisk walking and, in our experiments walking speed was fixed by the treadmill rather than being determined by the arousal state of the subjects. We note that the effects of exercise on neuronal feature selectivity and intracortical excitability that have been reported to date (Bullock et al., 2015, 2017; Neva et al., 2017) required a “somewhat hard” acute pedaling exercise of a type that the subjects in our study did not engage in. Therefore, perhaps surprisingly, our pupillometry measurements suggest that brisk walking did generate some level of arousal in our subjects; the increase of ∼34% in mean pupil area is almost identical to the increase caused by a transition from “rest” to the “low-intensity exercise” measured by Bullock et al. (2017), a change that the same group reports as causing a small but significant increase in mean P1 amplitude over occipital cortex in high-frequency nontarget trials (Bullock et al., 2015). We note that Bullock et al. (2017) reported the most significant behavioral and electrophysiological results when contrasting the rest and high-intensity exercise condition, whereas most of the differences that they measured in pupil size occurred between the rest and low-intensity conditions. Therefore, it is possible that pupil size is a highly nonlinear measure of exercise-driven arousal. Although the relatively gentle exercise that our subjects engaged in may have been sufficient to generate mild arousal, as indexed by pupil size, it may not have been energetic enough to cause measureable increases in neuronal responses.

Humans and mice may also differ in the level of neuronal modulation that can be driven by attention. Desynchronized states observed during active behavior in mouse visual cortex may be similar to attention-driven modulation in primates (Harris and Thiele, 2011), but it is possible that, in our studies, attentional drive was consistently high because subjects were able to direct their attention to the task regardless of the locomotion state. Could a constitutively high level of neuronal activity driven by attention have masked more subtle modulations linked to locomotion or arousal? We believe this is unlikely. The effects of attention on psychophysical contrast response functions are difficult to measure in humans (because attention is intrinsically linked to the psychophysical task), but when they are measured at a population level with EEG, early visual areas exhibit a moderate but significant increase in response, but not contrast gain, that is selective for neurons tuned to the stimulus (Lauritzen et al., 2010; Verghese et al., 2012). There would seem to be no reason why changes in sensitivity should be masked by such a modulation and, strikingly, we measured a significant reduction in SNR *R*_max_ for the unmasked probe during our EEG locomotion condition, indicating that we are able to measure a changes in this parameters, but that these changes are not in the direction predicted by mouse studies. Similarly, we measured a significant increase in *c*_50_ for the collinear masking condition when subjects were walking, again showing that this parameter was unlikely to have been driven to saturation by attentional effects. Nevertheless, it is possible that attention was masking activity in a subpopulation of neurons that would otherwise have been modulated by locomotion; further studies using EEG and a distractor task will be required to dissociate these effects fully.

Not all animal work finds a correlation between alertness and contrast sensitivity. Cano et al. (2006) and Zhuang et al. (2014), for example, reported a range of changes in layer 4 of the rabbit visual cortex that were correlated with alertness, including an increase in response gain and neuronal firing reliability, but no change in contrast sensitivity. Although our stimuli were different from those used by that group (specifically, we used flickering rather than drifting gratings), our psychophysical model fits are consistent with their findings, suggesting a locomotion-driven increase in *R*_max_. Although our EEG data (which largely reflect activity in V1) do not show such an effect, it is nevertheless possible that the mouse visual system is modulated by locomotion or arousal in a manner that is simply different from that found in other mammals. We believe that it would be valuable to measure the effects of locomotion on some of the other parameters studied in rabbits; in particular, orientation tuning for moving stimuli.

Two other potential confounds relate to the motion of the head during the locomotion condition. First, it is possible that head motion generates retinal slip causing the images to move across the retina slightly during each presentation. There is some evidence that retinal “blur” can degrade acuity at velocities >3°/s (Westheimer and McKee, 1975). Although the effect of retinal motion is more complex than a simple temporal integration (Burr, 1980), it is possible that center/surround stimuli are less well segregated in locomoting subjects and therefore overlap to some degree. This, in turn, might introduce a second, largely precortical and therefore untuned “overlay” masking effect (Petrov et al., 2005). We tested for the effects of poor image stabilization in the psychophysical experiments by introducing a third condition in which the images move rapidly during the 200 ms that they are presented. Thresholds in this condition were not significantly elevated relative to the “static” condition (Fig. 3) and, most importantly, there was no significant increase in untuned masking from the orthogonal mask condition. This is likely to be a conservative test for retinal slip. The motion of the stimuli was both brief (and therefore untrackable) and random (and therefore unpredictable), whereas motion on the retina introduced by imperfect fixation while walking would have a predictable motion trajectory. We therefore believe that retinal slip is not responsible for the increase in tuned surround suppression that we observed in the locomoting condition.

Finally, head motion also contributed to broadband instrument noise in the EEG signal. Could this have masked a spectrally localized increase in signal amplitude? Our data suggest not. Broadband noise increases the signal amplitude across all temporal frequencies, but the effect is strongly mitigated in SSVEP recordings because of the high level of signal averaging: noise is phase randomized and therefore averages rapidly to zero across multiple presentations. In comparison, the signal generated by the flickering stimulus is phase locked and is therefore unaffected by averaging across time bins. In our data, the mean response at the tagged input frequency was 0.47 μV in the stationary condition and 0.53 μV in the walking condition, an increase in magnitude of ∼0.06 μV. However, in comparison, the mean sideband amplitude increased from 0.03 to 0.19 μV, an increase of ∼0.13 μV. We expected broadband noise to be approximately equal across neighboring frequency bins. Our data therefore suggest that, if anything, the evoked signal amplitude decreased when subjects were locomoting and the increase in raw amplitude at 7 Hz was due to broadband noise (hence the apparent decrease in SNR seen in Fig. 6 and the corresponding decrease in *R*_max_ in Fig. 7).

Our results indicate that very low-level visual processing is not necessarily altered by locomotion in humans. However, it is also clear that periods of treadmill running can recalibrate the perception of egomotion in humans (Pelah and Barlow, 1996), presumably through a normalization mechanism that combines information about optic flow and motor function. The error minimization mechanisms that drive this normalization must be activated immediately when visual information fails to match that expected from the locomotion state (as in our experiments) and experiments with flow fields in more complex simulations have revealed signals relating to this sensory combination in mouse V1 (Keller et al., 2012; Saleem et al., 2013). We therefore hypothesize that it might be possible to measure large EEG signals relating to these errors in future experiments that present optic flow stimuli to locomoting subjects, ideally in a head-mounted display system that eliminates extraneous cues to egomotion.
